# The Control of Event-File Management

**DOI:** 10.5334/joc.187

**Published:** 2022-01-06

**Authors:** Bernhard Hommel

**Affiliations:** 1 Cognitive Neurophysiology, Department of Child and Adolescent Psychiatry, Faculty of Medicine, TU Dresden, DE; 2 Department of Psychology, Shandong Normal University, Jinan, China; 3 Institute for Psychological Research & Leiden Institute for Brain and Cognition, Leiden University, NL

**Keywords:** Action, Action and perception, Event cognition

## Abstract

According to the Theory of Event Coding, both perceived and self-produced events are coded by binding codes of the features of these events into event files. Here I argue that distinguishing between the actual binding process and the retrieval of event files is empirically difficult but theoretically important. As a first step towards disentangling these processes, I provide a brief overview of what the available evidence tells us with respect to the control of the binding process and the control of the retrieval process. I argue that there is not much evidence for selectivity of the binding process: Various kinds of stimuli and actions seem to be spontaneously integrated under various conditions, and there is increasing evidence that emotions, task instructions, and task context are coded into event files as well. On the other hand, there is increasing evidence for a high degree of selectivity of the retrieval process, suggesting that most if not all observations of effective impact on event files reflect an impact on retrieval, but not binding proper. I conclude by pointing out open questions and issues.

## Object files

What are the basic units of human cognition? According to the Theory of Event Coding (TEC: Hommel, Müsseler, Aschersleben & Prinz, 2001), people represent the (external or internal) events they experience in terms of their features, which they bind into event files (Hommel, 2004). The event-file concept is an extension of the original object-file concept introduced by Kahneman, Treisman, and Gibbs (1992), who claimed that processing visual objects leads to the integration and binding of these features into feature networks representing them. According to Treisman (1996), this integration serves what she considered the binding problem, which refers to an apparent inconsistency: Humans and other primates represent objects in a spatially distributed fashion, as indicated by the existence of numerous feature maps in the primate visual cortex (DeYoe & Van Essen, 1988), and yet, their conscious experience of these objects is not fragmented at all but appears to be unitary and integrated. The logic of this consideration has been rightly criticized (e.g., Cisek & Turgeon, 1999; van der Heijden, 1995), as it actually rests on a kind of Rylean category mistake and presupposes what Dennett (1991) has described as a Cartesian theater. Describing representation as distributed assumes a systems-level analytical view, by taking the human brain as the machinery underlying our mentally experience. Describing this experience as unitary assumes a personal-level view, which highlights the subjective implications of mechanistic processes. Given that I, as a person, do own the brain that houses all the representations that the mechanistic description refers to, there is actually no contradiction between the format of representation described from a systems level and the conscious experience of the thereby represented event. In a sense, it is the having of my brain that is sufficient for the integration. Hence, the theoretical motivation for introducing the object-file concept can be considered flawed, which implies that there is no logical necessity for a dedicated integration mechanism to account for coherent experience. In other words, coherent conscious perception does not *necessarily* presuppose binding. And yet, even though humans do not necessarily need to bind, numerous findings suggest that they nevertheless do. As demonstrated by Kahneman et al. (1992) and many that followed, visual information can be processed more quickly and more accurately if it appears in places that this information has occupied in a previous display: e.g., processing an X on the left and an O on the right is easier if one just saw an X the left and/or an O on the right. This suggests that letter identities are not stored completely independently from the corresponding locations, but that some links are formed between identity and location: feature binding that is.

## Event files

Objects are only some of the events that people process. Stimuli can be more complex and dynamic, and especially more temporally extended than static symbols appearing on a screen. And there is action, humans’ way to actively change their environment and generate stimulus information by themselves. While traditional information-processing accounts of human performance tended to keep perception and action apart logically, theoretically, and empirically, TEC argued that this approach may be misled. As Dewey (1896) had already emphasized, perception is more than passively registering some energy impinging one’s sensory surfaces. Indeed, before I can process a visual stimulus, I need to orient my body and my head towards this stimulus, fixate it with my eyes, and attend to it, and the entire act of acquiring the stimulus becomes part of the perceptual experience: e.g., I will perceive the stimulus as “left” if this is where I had to turn in order to sense it. The importance of active exposition is even more obvious with tactile perception, which hardly generates any useful information without actively exploring the perceived surface. Hence, perception does not only involve but actually relies on action. Reversely, action planning is assumed to be driven by the active anticipations of the action’s sensory consequences—as ideomotor theories have claimed before the beginnings of academic psychology (e.g., Harless, 1861), and actions are evaluated by the comparison between intended and actual sensory outcomes (for an overview, see Hommel, 2017). That is, perception is not just accompanying but actually driving action control.

According to TEC, the terms perception and action actually refer to the exact same sensorimotor activity: both perception and action operate by systematically moving one’s body in order to generate particular sensory information (or other transformations of one’s environment), only that the term perception emphasizes the generated sensory outcome (the ends) while the term action emphasizes the activity of generating it (the means; Hommel, 2009). One particularly interesting implication of this approach to perception and action is that actions are assumed to be represented in terms of their sensory consequences, which in turn means that actions may be represented exactly like objects are: in terms of their perceivable features. This theoretical move turns the sometimes implicit, sometimes explicit opposition between stimuli and responses, and their assumed functions in information processing, into a scenario in which both are considered to be pretty much the same stuff, to have the same organization, and to be processed according to the same operational logic. Among other things, that makes it much easier to understand how perception and action can interact, how people can learn doing things that they see, and in which sense stimuli can be compatible or incompatible with responses (Hommel et al., 2001). Following this reasoning, Hommel (1998) suggested that bindings may not just serve to represent objects, as suggested by Kahneman et al. (1992), but they may just as well represent actions and entire stimulus-action events—hence the term event file.

**Figure 1:**
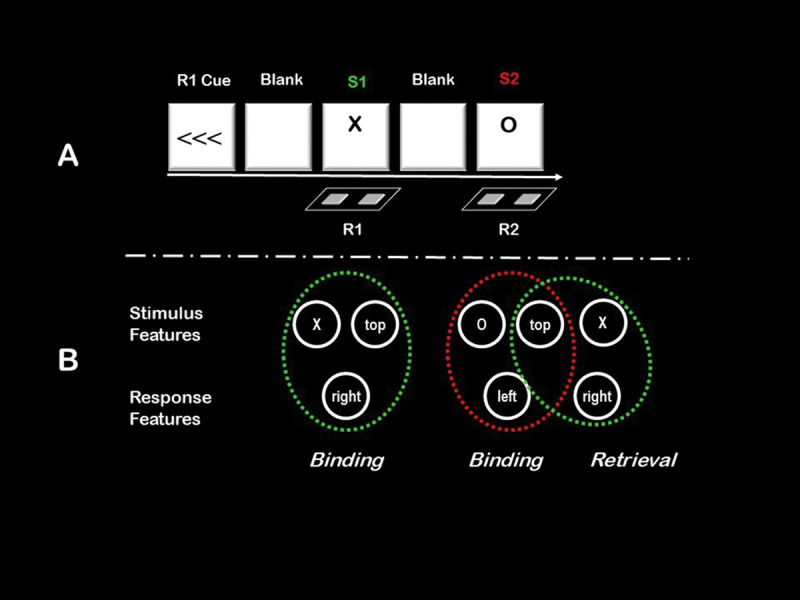
**A.** Basic components of the original event-file design introduced by Hommel (1998). Participants carry out two responses in each trial. A cue informs them whether the first response consists in pressing a left or a right key. They are to prepare the cued response but wait for the first stimulus (S1) to carry it out. Note that the features of S1 are entirely unrelated to the identity of R1, S1 merely functions as a go-signal. About a second or so later, the second stimulus (S2) appears. One feature of S2 signals the identity of R2, in this case the shape (the mapping in this example is X: right keypress; O: left keypress). **B.** The theoretical assumption of event-file theory. Presentation of S1 leads to the coding of the features of this stimulus, the shape “X” and the vertical “top” location in this example, and of the features of the corresponding response, “right” in this example. These three features, in addition to possible other (e.g., contextual or affective) features, are integrated into one event file, indicated in green. As soon S2 appears and R2 is selected and programmed, the corresponding features of S2 (“O” and “top”) and R2 (“left”) will be integrated the same way as those of S1 and R1, indicated in red. More importantly, however, is the fact that the combinations of S1/R1 and S2/R2 overlap with respect to the feature “top”. Even though this feature is actually irrelevant in this task, it will tend to retrieve event files that include the same feature, such as the one including the S1/R1 features (shown in green). This in turn will activate the included codes, which leads to a confusion regarding the shape and the response. Resolving this conflict takes time, which leads to worse performance in all conditions in which feature overlap between S1/R1 and S2/R2 exists but is incomplete—the so-called partial-repetition costs. Conditions with no overlap do not lead to this kind of retrieval and conditions with complete overlap do not lead to feature conflict. Note that this figure is very selective: processing S1/R1 and S2/R2 will also lead to the retrieval of feature-overlapping event files from previous trials. This is assumed to create further noise and code conflict, but given that appropriate research designs make sure that all feature combinations are balanced across trials, these kinds of conflict should not create a systematic bias of the outcomes.

The existence of event files has been demonstrated in various experimental tasks and designs (see Hommel, 2004, and Frings et al., 2020, for overviews), like the extension of the object-file design of Kahneman et al. (1992) to study stimulus-response bindings by Hommel (1998). In these event-file studies, participants are commonly presented with two stimulus-response pairs (see ***Figure 1***): a prime that consists of a particular stimulus (S1) and a particular response (R1), followed by a probe that consists of a stimulus (S2) and a response (R2) that either differ from S1 and R1, or repeat the stimulus but not the response, or vice versa, or repeat both stimulus and response. The orthogonal manipulation of stimulus and response repetition requires particular experimental tricks, like the pre-cuing of R1—without which the response could not be varied independently from the stimulus (for other techniques to deal with this problem, see Frings, Rothermund & Wentura, 2007, or Mayr & Buchner, 2006). Unsurprisingly, repeating stimuli and responses tends to induce simple priming effects: repetition speeds up performance. The more interesting observation in these tasks is that performance with complete repetition is commonly as good as performance with complete alternation, whereas repeating one element but alternating the other impairs performance substantially. Hence, there is a *partial-repetition cost* if some features of an event repeat while others change. This partial-repetition cost has been observed for numerous stimuli, responses, and stimulus features, as briefly touched in the next section, and it can be considered the hallmark of feature binding. Hence, experiencing a stimulus and a response in close temporal and spatial proximity somehow connects the representations of this stimulus and this response in such a way that experiencing a feature-overlapping event tends to retrieve not only the representation of the repeated feature of the event but also the feature(s) that previously accompanied that feature.

The fact that feature binding in these kinds of tasks generalizes over various kinds of features raises the question whether the binding principle can also be found for other kinds of stimuli, stimulus modalities, and actions. What is more, given the nature of the human brain, a feature code must consist of a set of neurons, neural connections, and/or neural communication (like synchrony for instance: Singer, 1994), and the actual coding of a feature must consist in the neural state produced thereby. If so, binding actually consists in the present and future coordination of neural states, which in turn raises the question whether “bindable” neural states are restricted to stimulus and response features in a narrower sense. For instance, the Jamesian account of emotion claims that the phenomenal experience of particular emotions results from the readout of internal states, which may refer to stimulus events, to one’s own actions, to one’s body posture, or other internal, interoceptively available states. All these states represent particular events, and the fact that some of them are actually internal (like the affective reaction to a stimulus) does not render them any less code-like and any less bindable than states representing a green circle, say. The same argument can be made for intentions, motivational states, or other states that accompany and, thus, contextualize event processing (Hommel, Pösse & Waszak, 2000; Spapé & Hommel, 2008). Indeed, as I will describe below, substantial evidence for the binding of various kinds of states has been reported.

Another reason to suspect that binding is not restricted to simple features, as represented in early feature maps of the human brain, and brief, almost reflex-like keypressing movements, relates to the coding nature of even the most low-level feature code. The information to which these codes react and which they therefore represent is commonly considered to be simple, direct, and basic, as compared to what is assumed to be categorial, integrative, or even symbolic (e.g., Fodor, 1975; Pylyshyn, 1984). It is a widely shared idea that the brain represents events in a hierarchically ordered representational system, in which direct representations at the lowest level are integrated by higher-order categories systems (Botvinick, 2008; Rosch et al., 1976; Vallacher & Wegner, 2012), and the hierarchy concept has also been applied to event files recently (Moeller & Frings, 2021). However, while the distinction between less integrative and more integrative levels is certainly easy to defend (which does not necessarily apply to the hierarchy assumption: e.g., Botvinick & Plaut, 2004), the distinction between simple and direct representations on the one hand and categorial representations on the other is not overly plausible and actually misleading, however. Note that even the simplest feature code in low-level, early cortical feature maps permits some level of variability over time and stimuli, which is a feature of every coding system that has limited resolution. Given that overlooking variability and differences in treating slightly different stimuli as the same is the defining feature of categorial representation, even the lowest representational level must be considered to be categorial to some degree. If so, there is no a-priori reason to doubt that more integrative categorial representations would not be showing the same operational principles than less integrative categorial representations. To anticipate, this is exactly what the available findings suggest.

The main question I would like to pursue in the following is how event files are managed. As I will explain in the next section, a closer look reveals that the results of event-file studies are not as easy to interpret as the first studies have suggested, which among other things makes it difficult to attribute the causes of individual differences and the impact of particular manipulations to specific processes. Disentangling these processes and the respective attributions requires, as I will explain, the distinction between the process of feature binding proper and the retrieval of feature bindings (Frings et al., 2020). A closer look also reveals that the factors that control the former are not identical with the factors that control the latter.

## Control of binding

The classical proofs of principle of binding phenomena concentrated on well-structured situations in which it was rather clear which of the presented information counted as the critical stimulus and what counted as response. Displays in object-file studies like that of Kahneman et al. (1992) and event-file studies like that of Hommel (1998) presented only one or few visual symbols to which participants responded by pressing one of very few keys. But our daily realities tend to be more complex and less well-controlled, which raises the question *what* is being bound under these circumstances and *when* or under which conditions is it being bound. Both questions can have various meanings and their answers vary accordingly.

### Components of event files

One of the possible *what* questions refers to the codes that can become ingredients of event files. The first studies were motivated by Treisman’s (1996) reference to visual feature maps in the primate brain and thus concentrated on simple features of visual stimuli, like shape, color, size, or motion—which so far have all shown comparable effects: repeating the exact combination of two given features or one feature and a response or alternating all of them allows for better performance than repeating some and alternating others. However, simple features coded in feature maps of the human brain do not exhaust the feature concept. Even maps within occipital cortex differ in complexity, with maps downstream the visual pathway coding for more complex features than the maps preceding them. Even further downstream we find “maps” that lose their spatial organization and combine various simple features to more complex aggregates representing parts of objects, or even entire objects, like faces and houses (Epstein & Kanwisher, 1998; Kanwisher, McDermott & Chun, 1997). While these representations are likely to be acquired through extended learning, humans can create ad-hoc categories that combine colorful combinations of features into arbitrary context-specific superfeatures according to which stimulus events can be sorted (Barsalou, 1983). Accordingly, one would expect that partial-repetition costs are not restricted to simple features coded in visual cortex, which is indeed what the evidence shows. For instance, binding effects have been demonstrated for complex features like faces and houses (Keizer, Colzato & Hommel, 2008a; Keizer et al., 2008b), words (Hommel & Müsseler, 2006), and abstract concepts (Singh, Frings & Moeller, 2017). Moreover, the human brain does not only code visual features in a distributed fashion but features of other modalities as well. Accordingly, binding studies have looked into audition and taction as well, and basically found the same kinds of binding effects (Zmigrod, Spapé & Hommel, 2009). Other studies have reported binding effects with various kinds of action: manual and vocal actions (e.g., Mayr, Möller & Buchner, 2011) or manual action sequences (Moeller & Frings, 2019). While these studies differ in methodology and in terms of the online or off-line character of the binding effects, the available evidence suggests that all kinds of features and actions can become ingredients of event files, and that their integration works in similar ways.

As explained, features are not restricted to the characteristics of perceived external events but may also refer to internal events. In fact, from the perspective of the cognitive system or the cortex, all representations of events in one’s environment are eventually internal states that are merely correlated with the actual physical events. From this perspective, there is no logical difference between the state of a color-sensitive neuron in occipital lobe and the state of neurons coding for one’s current state of arousal. As James (1884) has suggested, there is no logical difference between perceiving the world and perceiving oneself, as even our body is in some sense the environment that the cognitive system/the cortex live in and that they read out to generate information. Along the same lines, Barrett (2017) has claimed that phenomenally experienced emotions rely on the readout of neural states that are actually generated for other purposes, namely, for the regulation of basic bodily functions. If, thus, what we call emotional or affective states are, or reflect neural states that are logically comparable to other perceptual states, like neural activities in the occipital or temporal lobe, it is possible that the operational characteristics of binding also apply to such emotional or affective states.

If people code their emotions in terms of their perceptual consequences, as the Jamesian approach to emotion suggests, an event-coding account would imply that these perceptual codes may serve to represent both observed and produced events (Eder & Hommel, 2013; Lavender & Hommel, 2007). Indeed, numerous findings support this assumption, such as from studies on affective stimulus-response compatibility. The classical stimulus-response compatibility phenomena indicate that responses with particular features, such as pressing a left or right key, can be carried out faster and more accurately if they are signaled by stimuli that are sharing these features, so that a left-hand keypress, say, is faster if it is signaled by a stimulus on the left, rather than on the right of a display (Brebner, Shephard & Cairney, 1972). According to TEC or the Dimensional Overlap Model (Kornblum, Hasbroucq & Osman, 1990), this is because the overlap of features of the stimulus representation and the response representation leads to the automatic priming of the latter by the former, which supports performance in compatible, but impairs it in incompatible trials. There are also response-stimulus compatibility phenomena indicating that, for instance, having selected a left or right response facilitates the selection of a target in a corresponding location (Hommel & Schneider, 2002), supporting TECs claim that stimulus representations and action representations are equivalent and can interact in both directions. Interestingly, this positive compatibility effect turns into a negative effect if the action plan cannot yet be carried out but must be maintained, e.g., intending to press a left key impairs unspeeded identification of a left-pointing arrow (Müsseler & Hommel, 1997) and intending to say “left” impairs the identification of the word “LEFT” (Hommel & Müsseler, 2006). TEC attributes observations of this sort to “code-occupation” (Stoet & Hommel, 1999): binding a feature code to an active action plan occupies this code and interferes with using it for other purposes—until the plan is executed. Of particular interest, all these observations could be replicated with affective characteristics of stimuli and actions. For instance, performance is better when responding to words with positive or negative meaning by uttering words with corresponding connotations, such as “positive” and “negative” or “flower” and “cancer”, respectively (De Houwer & Eelen, 1998), and performing approach or avoidance responses interferes with the identification of affectively compatible positive and negative stimuli, respectively (Eder & Klauer, 2009). Hence, people seem to code their affective evaluation or reaction to stimuli or actions into the cognitive representations thereof, which in turn suggests that they treat these affective qualities like features. In other words, event files can include codes that represent emotions.

The possibility that event files include more than a particular stimulus and a particular response implies that the concept of an event file provides a unique opportunity to contextualize events, that is, to relate stimuli and actions to the context in which they were experienced. The advantage of contextualized representations lies in the fact that they permit more specific and more situationally adequate retrieval. Action control is often assumed to be governed by stimulus-response rules, but almost all rules make sense only with a particular intention and in a particular context. Learning to wait for a green light to cross a street can certainly be a good thing but applying the rule irrespective of one’s intention to actually cross the street and of the presence and proper functioning of a traffic light will likely to be dysfunctional. So how do we know, in the absence of explicit instruction, which actions to choose under which conditions? One theoretical approach consists in the formulation of not further specified stimulus-response associations. Given the number of stimuli and responses, and the different context conditions that determine which stimulus-response associations is currently appropriate, this implies a vast number of associations, which is hard to manage without a strict organizational regime. Some approaches have considered a hierarchical-sorting regime, under which simpler actions are considered to be ingredients of higher-order, more integrative representations, which in turn are the ingredients of even higher-order, even more integrative representations (Koechlin, Ody & Kouneiher, 2003; Schumacher & Hazeltine, 2016; Vallacher & Wegner, 2012). However, how these hierarchical systems are actually constructed and implemented, and where they come from in the first place has not yet been theorized about, and the fact that events can be verbally described in a hierarchical fashion may reveal more about the way of thinking of the describing individual than about the operational characteristics of the described system (Danziger, 1997). In fact, complex structures of actions that are commonly described in a hierarchical language, like preparing tea, can be easily modeled in a completely heterarchical fashion (Botvinick & Plaut, 2004).

The non-selectivity of event representation through event files, which considers even explicitly defined non-targets (Frings & Rothermund, 2011, 2017) and the affective implications of both the stimuli and the actions involved, provides an attractive and parsimonious theoretical alternative if only two assumptions are combined. For one, event files are assumed to be retrieved through feature overlap: as the partial-repetition costs show, facing an event with one or more features that are shared by already stored event files leads to the retrieval of files with sufficient overlap, with a stronger emphasis on features of currently relevant dimensions (Memelink & Hommel, 2013)—an issue I will get back to in the next section. For another, TEC and the ideomotor tradition assume that actions are represented in terms of their anticipated features. This means that goals can be considered bundles of features describing an intended effect (Hommel, 2021), which in turn will serve as selection criteria (Hommel & Wiers, 2017). This implies that action selection will always be contextualized in promoting event files that overlap with both, the current goal and the current context. In other words, storing context information in event files may lead to an automatic contextualization of action selection, without assuming any higher order system that is responsible for this functionality.

Increasing evidence indeed suggests that task-related context information becomes integrated into event files. For instance, if participants are to switch between reading the word and naming the picture of Stroop-like word-picture compounds, they have a harder time to switch to word reading if the present compound had been picture-named before, even if that happened more than 100 trials ago and only once (Waszak, Hommel & Allport, 2003). Along the same lines, stimuli in a switching task were found to allow for better performance if they were associated with the same stimulus-response mapping three trials ago (Pösse, Waszak & Hommel, 2006), and aftereffects of binding are reduced in size if irrelevant features of the context change, like the voice in which Stroop-like distractors are presented (Spapé & Hommel, 2008). These and other observations strongly suggest that “cognitive representations of perceived and produced events (i.e., perception and action codes) are contextualized by integrating them with codes of events they accompany” (Hommel et al., 2000, p. 227).

### Conditions for integration

One *when* question refers to the circumstances under which components are integrated into event files. Various studies have systematically manipulated the amount of attention needed to process especially stimulus components, but none suggested any important role of attention. For instance, Hommel (2005) manipulated the attentional relevance of stimulus components in various ways across eight experiments, by rendering them entirely irrelevant, by making them follow (rather than precede) the response, by putting them into competition with task-relevant stimuli, and by having participants to suppress the response it accompanied—but there was no indication of any impact on the binding process. So far, there seem to exist only two exceptions to this general picture of a high degree of non-selectivity in binding features. First, there are some indications that more salient stimuli might be associated with stronger binding-related effects both in stimulus-response binding (Schmalbrock, Laub & Frings, 2021) and in action-effect binding (Dutzi & Hommel, 2009). Second, task-relevance of stimulus dimensions has played an important role from the very first study on (Hommel, 1998)—where partial-repetition costs were more pronounced for the relation between the response and features of the dimension that defined S2 (shape or color) than for the relation between the response and features of other, task-irrelevant dimensions (color and shape, respectively). Task-relevance effects have also been shown for responses (Hommel, 2007), in the sense that bindings between stimulus location and the response are much more pronounced if the response is also spatially defined than if it is not. Relatedly, action-effect binding is stronger for relevant than for irrelevant action features (Mocke, Weller, Frings, Rothermund & Kunde, 2020). As discussed in the next section, there are reasons to believe that task-relevance relates to retrieval, rather than binding, and there is some evidence that the same may be true for salience: Laub, Frings, and Moeller (2018) observed that distractors have a higher potency to retrieve previously bound responses if they are perceptually sticking out more (i.e., if they are easier to discriminate from the target) at retrieval.

Another version of the *when* question refers to spatial segmentation. Situations that involve more than one stimulus, such as a real-world visual scene, raise the question how one defines what a stimulus is. Psychology has not yet achieved a straightforward answer to this complicated question, given that people have some flexibility with respect to the spatial and temporal segmentation of information into functional stimulus units. This issue has been raised by Gestalt psychology, which tried to identify at least the most obvious principles that people seem to use when constructing stimuli from a rich scene: common fate, grouping, Prägnanz, continuity, connectedness, and more. People have also considerable flexibility in coding complex scenes into either more local or more global informational units, as shown by Navon (1977), and there is evidence that binding effects are sensitive to this flexibility. For instance, van Dam and Hommel (2010) observed that orientation and color features are bound even if they belonged to different objects as long as these objects were presented in the same location (and thus overlapped in space), but not if the objects they belonged to occupied close but separable locations. Along the same lines, Frings and Rothermund (2011, 2017) found that the repetition or alternation of the relationship between distractors and responses matters more, or even only if the display presents distractors as part of a perceptual group containing the target. Importantly, this is not due to a tight limitation with respect to the number of stimuli that can be bound into an event file: Hommel (2002) presented participants with four targets at the same time, and tested whether retrieving information about the shape of these targets up to four trials later would also reactivate information about the location of the target. This was the case, suggesting that four event files could be created and concurrently maintained, an observation that also fits with findings of object-file studies (Scholl & Pylyshyn, 1999).

Events do not only need to be segmented in terms of space, but also in terms of time. As pointed out by Barker (1963), facing our world implies being exposed to a continuous stream of information without defined beginnings and endings. And yet, if people are asked to segment events in time, like episodes in a movie, they are remarkably consistent, suggesting that there are particular markers that allow identifying episodes of a particular meaning (Newtson & Engquist, 1976; for an overview, see Stränger & Hommel, 1996). Predictability seems to play an important role in this identification. Zacks and colleagues (2007) have suggested that people monitor online informational streams and try to predict what’s coming next. If this prediction fails to some degree, they reset their working memory and start constructing a new episode. However, people are not only very consistent in episode identification when having no particular instruction, but they can also be instructed to use a finer or coarser grain size in this identification (Newtson, 1973)—very similar to the flexibility in attending more local or more global aspects of complex stimulus arrangements (Navon, 1977). That binding can be affected by this flexibility has been suggested by Akyürek, Riddell, Toffanin, and Hommel (2007; Akyürek, Toffanin & Hommel, 2008). They used an attentional blink task, in which participants are exposed to streams of symbols presented in very quick succession. When attending to targets presented among distractors (e.g., digits among letters), performance for the second of two targets is often poor if the second target appears too soon (about 100–500 ms) after the first. Interestingly, however, this so-called attentional blink is absent (i.e., performance is perfect) if the second target appears right after the first, i.e., with a temporal lag of 1. This lag-1-sparing effect has been attributed to the integration of the two targets into one episode (or event file), which fits with the observation that perfect performance often comes with the inability to report the actual sequence of the two targets (Akyürek & Hommel, 2005). Akyürek and colleagues (2007) reasoned that the probability of target-target integration might depend on the expectation of a fast-changing or slow-changing visual stream. They used an illusion to introduce the expectation of a fast-changing stream in one group of participants and of a slow-changing stream in another group, hypothesizing that the former should adopt a smaller temporal integration window, and thus be less likely to integrate the two successive targets, whereas the latter should adopt a larger temporal window, which should make integration more likely. Indeed, perfect performance coming with order confusions (hallmark of integration) was more likely in the slow-expectation group. Even more interestingly, EEG recordings related to the processing of the second target showed two distinct components in the fast-expectation group that were entirely absent in the slow-expectation group. This suggests that expecting a fast rate of information increases the probability that people code the same two pieces of sequential information as belonging to separate events and open a new event file when the second is presented.

Taken altogether, there is increasing evidence that the control of feature binding is rather indirect. Integration seems to be highly non-selective with respect to the task-relevance and nature of the integrated features, be they related to external states of affairs, like stimuli, responses, or their effects, or to internal states, like those driving emotions or task representations. However, integration does depend on how information is segmented in time and space, and the temporal and spatial overlap of information belonging to the same event. The definition of this overlap does not seem to be fixed and invariant, but rather seems to depend on how tightly a given person is currently integrating, how finely grained the current integration window is. In other words, there is some variability with respect to how broadly an event is defined but all elements falling into the current definition seem to be bound.

## Control of retrieval

Most tasks that study binding effects have a certain sequential logic. Participants are presented with events that are assumed to induce a particular binding, and with another event the processing of which is assumed to be affected by the induced binding. This holds for the object-file paradigm of Kahneman et al. (1992), in which a prime display contains symbols in particular locations, with the expectation that this induces the creation of symbols-location bindings. And for Hommel’s (1998) event-file paradigm, in which the co-occurrence of a stimulus and a response is assumed to induce a stimulus-response binding that is retrieved by presenting a similar or identical stimulus and/or having participants perform a similar or the same response (see ***Figure 1***). Other paradigms even encourage or require participants to keep a particular binding active (such as a plan to carry out a particular action) while processing another stimulus, response, or stimulus-response combination that is assumed to be affected by that binding (e.g., Müsseler & Hommel, 1997; Stoet & Hommel, 1999; see Hommel, 2004). If these kinds of manipulations have a particular impact, so that for instance responses to a particular stimulus are delayed if this stimulus has just been paired with another response (Hommel, 1998), the conclusion is straightforward: a particular binding was apparently created and retrieved, resulting in the observed findings. However, the absence of such effects is more difficult to interpret. Given that both binding and retrieval are necessary requirements for any result to emerge, at least in behavioral studies, null findings related to particular conditions or manipulations might be due to the absence of binding, of retrieval, or of both. More specifically, the absence of a behavioral effect cannot be taken as evidence for the absence of binding, nor can the presence of an effect of a particular manipulation be taken as a demonstration that binding was affected.

The importance of this distinction between binding and retrieval was only recently emphasized by Frings and colleagues (2020), because these two possible targets of manipulations have not always been sufficiently distinguished in the research on object and event files. However, the bulk of the evidence coming from studies on individual differences and particular interventions is much easier to make sense of if interpreted in terms of retrieval rather than binding effects. For instance, Hommel, Kray, and Lindenberger (2011) studied stimulus-response binding in 9-10-year-old children, and in young and old adults in a classical event-file task. Aftereffects of bindings were significantly more pronounced in children and the elderly group, as compared to young adults. Taking this observation to indicate an effect of feature binding proper would suggest that participants in the younger and the older group would be better in integrating information than young adults. Such a conclusion would be counterintuitive and inconsistent with various findings from lifespan research, like the observations of Hommel, Li, and Li (2004) in a visual search task. Search performance is known to be less efficient when searching for feature conjunctions (e.g., searching for a filled circle among unfilled circles and filled squares) than for simple features (e.g., searching for a filled circle among unfilled circles), which is commonly attributed to the need to integrate the features of the stimuli before being able to judge whether a conjunctively defined target has been found (Treisman & Gelade, 1980). A related finding is that performance decreases with an increasing number of stimuli in a display (the set size) when searching for feature conjunctions but not when searching for simple features, suggesting that conjunction search imposes extra processing costs for each single display element (Treisman & Gelade, 1980). Hommel et al. (2004) had participants from 6 to 89 years of age search for simple features and feature conjunctions. As expected, the extra costs associated with conjunction search were substantially more pronounced in children and in the elderly, as compared to young adults, and the same was true for the effect of set size in conjunction search.

These findings demonstrate that children and older adults have a rather hard time integrating features, which is the opposite of what a binding-interpretation of Hommel et al.’s (2011) observations would imply. In contrast, an interpretation of these observations in terms of retrieval makes much more sense. The classical event-file task, like most tasks investigating binding effects, does actually not require or reward the retrieval of any binding. The combination of features is commonly fully random, so to discourage participants from intentionally recalling the previous feature combination. On the one hand, this methodological characteristic provides strong evidence that feature binding is a spontaneous process that accompanies regular stimulus processing and response planning. On the other hand, however, it means that participants do not benefit at all from retrieving any object or event file. Accordingly, differences in the tendency to retrieve feature bindings in these kinds of tasks can be taken to indicate a lack of control over retrieval. Logically, no retrieval would be necessary, so that it would be a good strategy to avoid the retrieval of bindings at all. Indeed, individuals with high scores in the Raven matrix, an intelligence test, were found to show significantly smaller binding aftereffects than individuals with lower scores (Colzato, van Wouwe, Lavender & Hommel, 2006). This in turn would suggest that the stronger retrieval found for children and elderly actually indicate less efficient control over retrieval, which is consistent with numerous findings from lifespan studies (e.g., Li, Lindenberger, Hommel, Aschersleben, Prinz & Baltes, 2004). An interpretation of larger aftereffects of binding in terms of impaired retrieval is also much more consistent with other observations of individual differences. For instance, Zmigrod, de Sonneville, Colzato, Swaab, and Hommel (2013) found larger aftereffects of binding in children with autistic spectrum disorder than in healthy controls. Given that autism and related disorders are known to be associated with severe difficulties in feature integration, a binding interpretation would run into substantial theoretical problems, whereas interpreting aftereffects in terms of a lack of control over spontaneous retrieval makes perfect sense.

A more direct attempt to disentangle binding from retrieval effects was entertained by Hommel, Memelink, Zmigrod, and Colzato (2014), who made use of the already mentioned observation that binding-related effects are more pronounced for features of dimensions that are explicitly task-relevant (e.g., Hommel, 1998). Logically, this effect of task-relevance might be due to binding (i.e., features of a relevant dimension might be more likely to enter the binding process) or retrieval (i.e., features of a relevant dimension might be more strongly contribute to retrieval), or both. To disentangle these two processes, Hommel and colleagues (2014) varied the relevant feature dimension (color and shape) randomly from trial to trial, and they did so either before presentation of S1 or before presentation of S2. As a consequence, the relevant dimension could be the same during feature binding (S1-R1 processing) and feature retrieval (S2-R2 processing) or different. If relevance would have an impact on binding, this would only lead to a stronger effect of the relevant binding if the relevant dimension is signaled before S1 presentation, but not if it appears after the binding is completed. As it turned out, the time point of presenting the dimension cue did not matter, suggesting that it is not binding, but retrieval that produces the relevance effect. In other words, retrieval might be selective with respect to the integrated features while binding is not.

An interpretation in terms of retrieval control is further supported by outcomes of two neurofeedback studies (Keizer, Verment, & Hommel, 2010a; Keizer, Verschoor, Verment, & Hommel, 2010b). These studies were based on the idea that feature binding may rely on neural communication in the gamma frequency range (Singer, 1994). The original aim was to use neurofeedback to teach participants to increase their neural activities in this frequency band in their occipital lobe, so to support the binding of visual features. Participants were thus presented with electrophysiologically derived feedback of gamma activity in sensors over their occipital lobe, to see whether this would increase the aftereffects of feature binding in an event-file task. Two observations indicated that the original aim was misled, however. First, there were indications that the increase of gamma activity over the occipital cortex was actually achieved by frontal areas, and indeed using feedback from sensors over the frontal cortex produced the same learning effects. Second, even though the neural feedback manipulation was successful in teaching participants to increase gamma activity, increases in this activity were associated with smaller, rather than larger partial-repetition costs in the behavioral task. Both observations suggest that the manipulation did not affect binding processes proper but retrieval, apparently organized by frontal areas and apparently making retrieval more selective. Indeed, the aftereffects of bindings between the relevant stimulus feature and the (also relevant) response were more or less unaffected by learning, while bindings involving irrelevant stimulus features decreased over time. Also of interest was the observation that the learned increase of gamma activity was associated with an increase in the intelligence score. Remember that a higher score was found to predict smaller partial-repetition costs (Colzato et al., 2006), suggesting that the intelligence score is associated with processes that control the degree to which incoming information gets access to, and can reactivate previously bound information. In other words, intelligence must have something to do with the selectivity of retrieval. Converging evidence for this assumption is the observation that the neural feedback training did not only reduce partial-repetition costs but also improved performance in a long-term memory test—i.e., a measure of explicit retrieval (Keizer et al., 2010b).

Further evidence for a role of the frontal cortex comes from a tDCS study of Zmigrod, Colzato, and Hommel (2014). Both anodal and cathodal stimulation of the right dorsolateral prefrontal cortex induced a significant increase of partial-repetition costs as compared to a sham condition and to the stimulation of the left dorsolateral prefrontal cortex. Given that anodal and cathodal stimulation had the same effect rules out the standard interpretation of these two conditions as facilitating versus interfering with the operation of a particular area or functional system. Rather, any effective stimulation seems to have interfered with operations of the targeted area, suggesting that it houses processes responsible for selective retrieval of event files.

## Conclusions

Taken altogether, the bulk of the evidence suggests that the process of binding, the integration of stimulus and response features into event files, is highly non-selective with respect to the integrated features and also considers codes representing the current context, the task, and even affective states. Retrieval, in contrast, seems to depend on the task-relevance and the individual ability or disability to fine-tune the retrieval process. These observations call for a more systematic differentiation between binding and retrieval processes, and a more systematic theoretical treatment of these processes. While there seems to be a general tendency of the available findings, quite a bit of my argumentation rests on post-hoc re-interpretations of available findings and mere plausibility, and various empirical details still need to be sorted out. For instance, some of the observations of changes in partial-repetition costs under some experimental conditions or in inter-individual comparisons refer to bindings between task-relevant features and the response, while others refer to bindings between task-irrelevant features and the response. Bindings between task-relevant and task-irrelevant stimulus features are rarely considered and if they are, they often behave differently than stimulus-response bindings (e.g., Colzato, Erasmus & Hommel, 2004; Colzato, Fagioli, Erasmus & Hommel, 2005; Colzato, Warrens & Hommel, 2006; Giesen & Rothermund, 2014). Finally, it would be useful and important to better understand how the implicit retrieval processes indicated by partial-repetition costs obtained with standard event-file paradigms relate to explicit memory retrieval.
